# An *AGAMOUS* intron‐driven cytotoxin leads to flowerless tobacco and produces no detrimental effects on vegetative growth of either tobacco or poplar

**DOI:** 10.1111/pbi.12581

**Published:** 2016-06-20

**Authors:** Wei Li, Wei Hu, Chu Fang, Longzheng Chen, Weibing Zhuang, Lorenzo Katin‐Grazzini, Richard J. McAvoy, Karl Guillard, Yi Li

**Affiliations:** ^1^ Department of Plant Science and Landscape ArchitectureUniversity of ConnecticutStorrsCTUSA; ^2^ Institute of Vegetable CropsJiangsu Academy of Agricultural SciencesNanjingChina; ^3^ College of Horticulture and State Key Laboratory of Crop Genetics and Germplasm EnhancementNanjing Agricultural UniversityNanjingChina

**Keywords:** flowerless, neuter (stamenless and carpel‐less), *Populus*, transgene flow, *AGAMOUS*, Intron

## Abstract

Flowerless trait is highly desirable for poplar because it can prevent pollen‐ and seed‐mediated transgene flow. We have isolated the second intron of *PTAG2*, an *AGAMOUS* (*AG*) orthologue from *Populus trichocarpa*. By fusing this intron sequence to a minimal *35S* promoter sequence, we created two artificial promoters, *fPTAG2I* (forward orientation of the *PTAG2* intron sequence) and *rPTAG2I* (reverse orientation of the *PTAG2* intron sequence). In tobacco, expression of the β‐glucuronidase gene (*uidA*) demonstrates that the *fPTAG2I* promoter is non‐floral‐specific*,* while the *rPTAG2I* promoter is active in floral buds but with no detectable vegetative activity. Under glasshouse conditions, transgenic tobacco plants expressing the *Diphtheria toxin A* (*DT‐A*) gene driven by the *rPTAG2I* promoter produced three floral ablation phenotypes: flowerless, neuter (stamenless and carpel‐less) and carpel‐less. Further, the vegetative growth of these transgenic lines was similar to that of the wild‐type plants. In field trials during 2014 and 2015, the flowerless transgenic tobacco stably maintained its flowerless phenotype, and also produced more shoot and root biomass when compared to wild‐type plants. In poplar, the *rPTAG2I::GUS* gene exhibited no detectable activity in vegetative organs. Under field conditions over two growing seasons (2014 to the end of 2015), vegetative growth of the *rPTAG2I::DT‐A* transgenic poplar plants was similar to that of the wild‐type plants. Our results demonstrate that the *rPTAG2I* artificial promoter has no detectable activities in vegetative tissues and organs, and the *rPTAG2I::DT‐A* gene may be useful for producing flowerless poplar that retains normal vegetative growth.

## Introduction

Transgenic technologies provide a powerful tool to improve poplar for herbicide resistance, enhanced drought, cold and salt tolerance, increased growth rate and improved processing and end‐use characteristics (Tzfira *et al*., 1998; Ye *et al*., 2011). Even though many field trials for transgenic poplar traits have been conducted, none has been commercialized in the United States or other developed countries. One major concern obstructing the deregulation of transgenic poplar is the undesirable spread of transgenes to native ecosystems, known as transgene flow (Strauss *et al*., 2009).

Floral propagules are important vehicles of transgene flow. Pollen‐ and seed‐mediated transgene flow is difficult to control (Heuberger *et al*., 2010; Luo et al., 2007). It has been reported that pollen and seeds from poplar (*P. trichocarpa*) can be easily dispersed over long distances via wind, increasing the risk potential for transgene flow (DiFazio *et al*., 2012; Kausch *et al*., 2010; Oliver, 2004; Slavov *et al*., 2009; Zhang *et al*., 2012). In addition, pollen from poplar can induce allergic reactions in a large segment of the population (Çelik *et al*., 2005). As vegetative propagation is preferred over sexual reproduction for most commercial cultivars of poplar, engineering flowerless poplar could provide an effective means to address pollen‐ and seed‐mediated transgene flow and allergy problems (DiFazio *et al*., 2012; Kausch *et al*., 2010; Oliver, 2004; Zhang *et al*., 2012). Furthermore, with reproductive organs eliminated, more photosynthetic products can be used for vegetative growth, likely leading to improvement of biomass production.

A flowerless phenotype could be achieved using a flower‐specific promoter to drive expression of a cytotoxic gene (Skinner *et al*., 2003; Wei *et al*., 2007). However, none of the ‘flower‐specific’ promoters tested has been shown to be flower‐specific in poplar plants, because when used to control expression of a toxin gene, the resulting transgenic plants displayed reductions in vegetative growth (Brunner *et al*., 2000; Igasaki *et al*., 2008). For instance, Elorriaga *et al*. (2014) reported that all transgenic poplar plants, expressing a barnase gene driven by the *TA29* promoter, a tapetum‐specific promoter cloned from tobacco, exhibited retarded vegetative growth. Losses of the floral specificity for these promoter sequences, such as the tobacco pollen‐specific *TA29* promoter, in poplar demonstrate that floral specificity of promoter sequences from one plant species may not be retained in a different species due to evolutionary divergences. Therefore, the use of a floral‐specific promoter from poplar may be necessary for engineering flowerless poplar with no negative effects of its vegetative growth.

*AGAMOUS* (*AG*), a gene coding for a MADS protein, is expressed in the third and fourth whorls of *Arabidopsis* flowers and is essential for floral meristem determinacy (Bowman *et al*., 1991). A 4‐kb enhancer sequence located within the second intron of *AG* was found to influence *AG* expression during early stages of flower development of *Arabidopsis* (Deyholos and Sieburth, 2000). Chimeric promoters derived from the second intron of *Arabidopsis AG* have been used to drive expression of the cytotoxic gene *Diphtheria toxin A (DT‐A)* in *Arabidopsis* plants. Flowerless *Arabidopsis* plants have been achieved with no detrimental impacts on vegetative growth (Liu and Liu, 2008). However, Wang *et al*. (2008) reported that using the same promoter in tobacco plants produced imprecise and inefficient floral organ ablation. These results suggest that *AG* intron‐derived chimeric promoters from *Arabidopsis* or other species may not maintain their floral specificity and effectiveness if used in poplar. Therefore, it is highly desirable to isolate a floral‐specific gene promoter from poplar species, in order to develop a flower‐specific promoter for producing flowerless poplar plants. Here, we report the use of the second intron sequence from an *AG* homolog (*PTAG2*) cloned from *P. trichocarpa* to control expression of a β‐glucuronidase gene (*uidA*) and a toxin gene (*DT‐A*) in both tobacco and poplar. We demonstrate that the reverse orientation of the *PTAG2 *second intron sequence confers floral dominant expression with no detectable vegetative activity, and toxin gene expression, under the control of this sequence, leads to a flowerless phenotype in tobacco. This construct also had no observable negative effects on biomass in either tobacco or poplar.

## Results

### The reverse‐oriented second intron of *PTAG2* conferred flower‐specific expression

**Figure 1:**
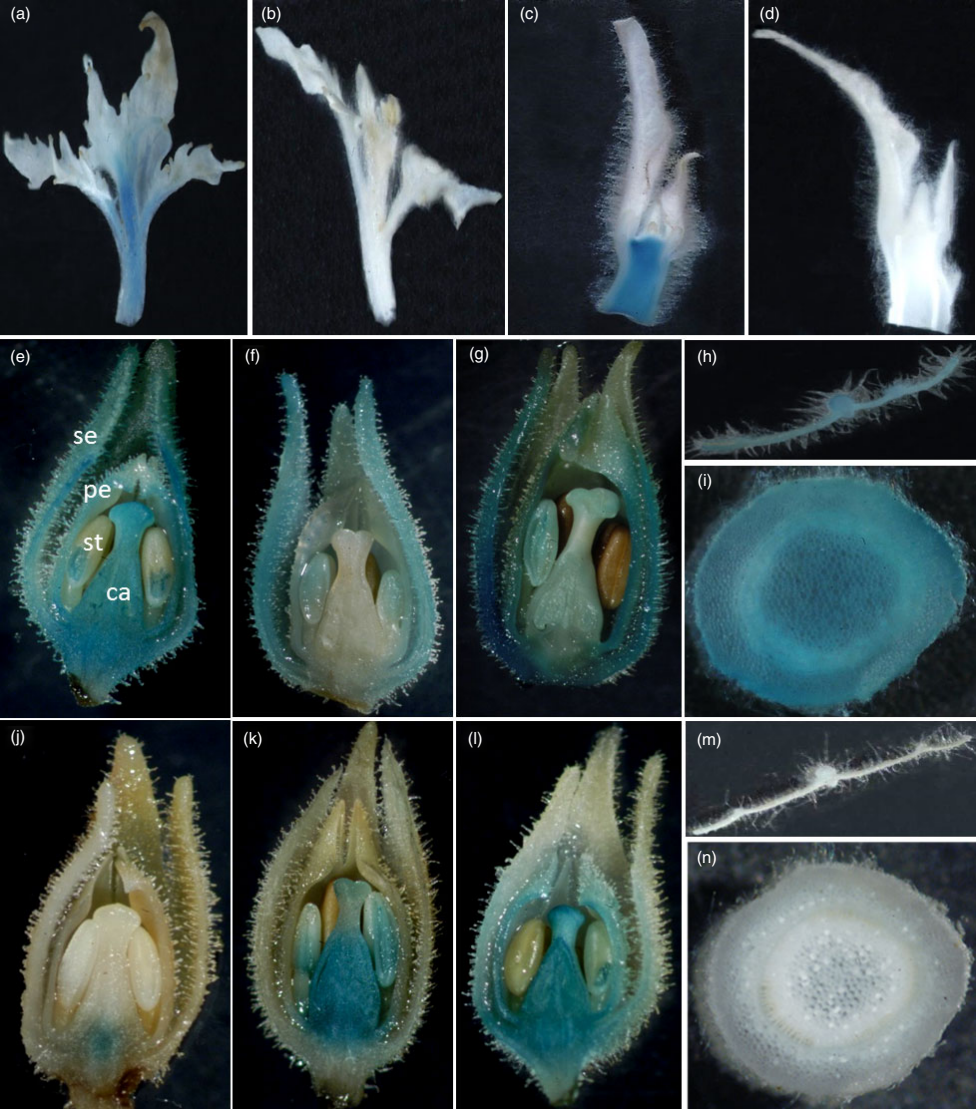
Histochemical staining of GUS activity in *fPTAG2I::GUS* and *rPTAG2I*::*GUS* poplar and tobacco. (a, b) Shoot apices from 2‐month‐old transgenic poplar plants harbouring *fPTAG2I::GUS* (a) and *rPTAG2I::GUS* (b). (c, d) Longitudinal sections of shoot apices from five‐week‐old glasshouse‐grown transgenic tobacco plants harbouring *fPTAG2I::GUS* (c) and *rPTAG2I::GUS* (d). (e–g) Longitudinal sections of floral buds from three different 4‐month‐old *fPTAG2I::GUS* tobacco lines showed varying levels of *fPTAG2I* promoter activity. (h, i) Cross sections of leaf (h) and stem (i) from 4‐month‐old *fPTAG2I::GUS* tobacco exhibited GUS activity. (j–l) Longitudinal sections of floral buds from three different 4‐month‐old *rPTAG2I::GUS* tobacco lines showed varying levels of *rPTAG2I* promoter activity. (m, n) Cross sections of leaf (m) and stem (n) from 4‐month‐old *rPTAG2I::GUS* tobacco exhibited no GUS activity. se: sepal; pe: petal; st: stamen; ca: carpel.

To test the flower specificity of the *PTAG2 *second intron, we transformed tobacco and poplar using *A. tumefaciens* strains hosting *fPTAG2I::GUS*, and *rPTAG2I::GUS* genes. Both transgenic poplar and tobacco plants were assayed with histochemical staining of GUS activity in leaf, stem, and apical meristem tissues. At vegetative stages, *fPTAG2I::GUS* expression in both poplar and tobacco plants were detected in stem and vegetative apical meristem tissues (Figure 1a,c). At reproductive stages, *fPTAG2I::GUS* tobacco plants displayed GUS activity in floral organs (Figure 1e,f,g) and also vegetative tissues (Figure 1h,i). For *rPTAG2I::GUS* transgenic poplar and tobacco lines, none of them had any detectable GUS activity in vegetative tissues before flowering (Figure 1b,d). At flowering stages, *rPTAG2I::GUS* tobacco lines showed GUS activity in floral organs with three expression patterns: (i) expression in carpel (Figure 1j); (ii) expression in stamen and carpel (Figure 1k); and (iii) expression in sepal, petal, stamen and carpel (Figure 1l). None of *rPTAG2I::GUS* tobacco lines showed any detectable GUS activity in vegetative tissues at reproductive stages (Figure 1m,n). The histochemical staining of the GUS activity demonstrates that both the forward and reverse orientations of the *PTAG2* second intron are highly active in floral organs, but the forward orientation sequence (*fPTAG2I*) is active in vegetative organs while the reverse orientation sequence (*rPTAG2I*) has no detectable vegetative activity. We chose *rPTAG2I* promoter for further experiments.

### Floral organ ablation directed by the *rPTAG2I* chimeric promoter in glasshouse‐grown tobacco plants

**Figure 2:**
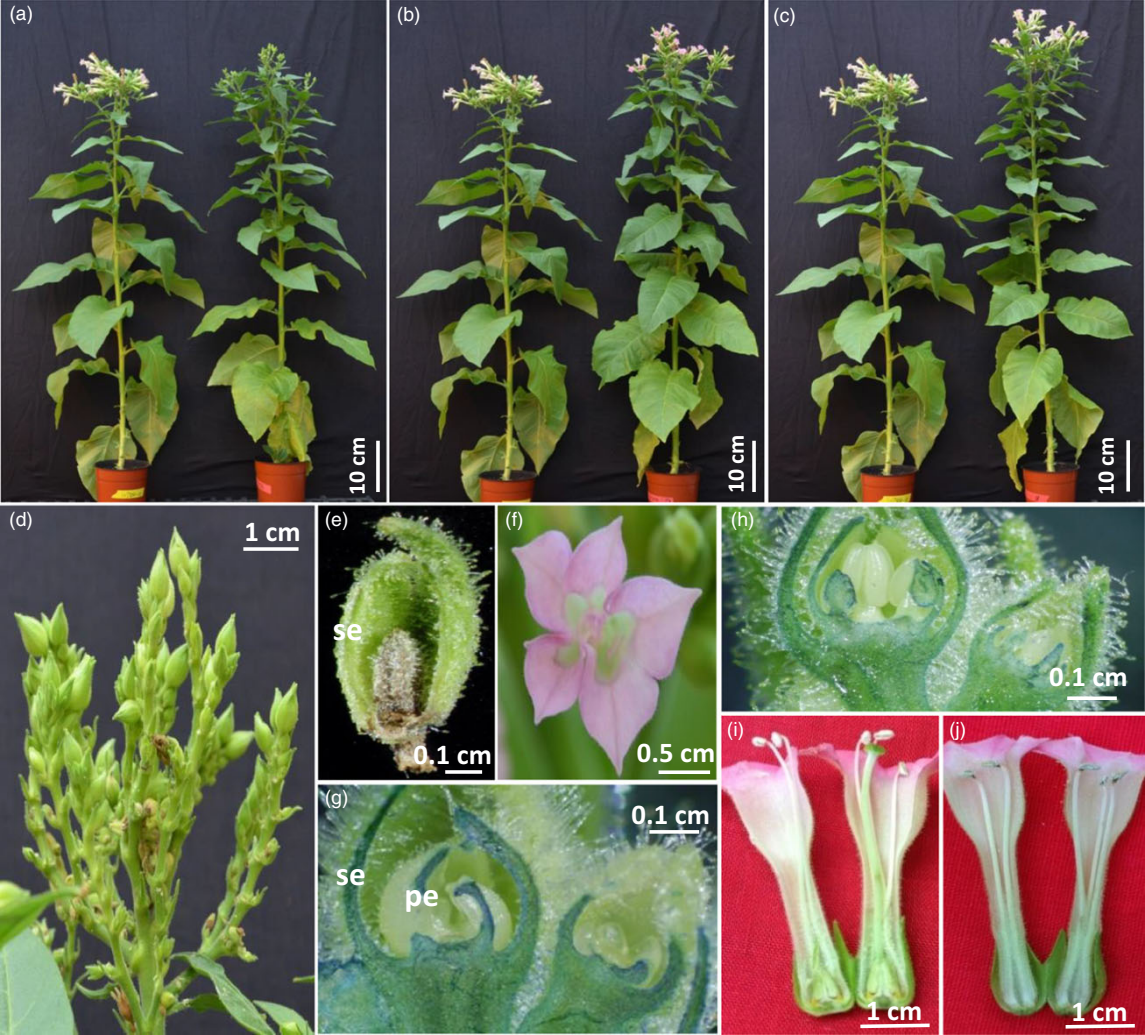
Floral organ development of glasshouse‐grown *rPTAG2I::DT‐A* tobacco plants. (a–c) Four‐month‐old *rPTAG2I::DT‐A* transgenic plants (right) alongside wild type plants (left). Transgenic plants displayed normal vegetative growth and one of three floral ablation phenotypes: flowerless (*rPTAG2I::DT‐A‐*Line‐8) (a)*,* neuter (stamenless and carpel‐less, *rPTAG2I::DT‐A*‐Line‐27) (b), or carpel‐less (*rPTAG2I::DT‐A*‐Line‐35) (c). (d) A closer look at the floral buds from *rPTAG2I::DT‐A‐*Line‐8. All floral buds were aborted before reaching stage 9 of floral development. (e) A longitudinal section of a floral bud from (d) showing petals, stamens and carpel were aborted. (f) A flower with no stamens or carpels (*rPTAG2I::DT‐A‐*Line‐27). (g) A longitudinal section of floral buds from *rPTAG2I::DT‐A‐*Line‐27 having stamens and carpels aborted. (h) A longitudinal section of floral buds from *rPTAG2I::DT‐A‐*Line‐35 having carpels aborted. (i, j) A longitudinal section of a flower from *rPTAG2I::DT‐A‐*Line‐35 (j) having carpel aborted when compared to that from a wild type plant (i). se: sepal; pe: petal.

The *rPTAG2I* chimeric promoter we constructed was used to drive a *Diphtheria toxin A (DT‐A)* gene, coding for a ribosome inactivating protein (Palmiter *et al*., 1987). Among 51 *rPTAG2I::DT‐A* tobacco lines produced, 48 lines exhibited floral ablation phenotypes while the other three lines displayed a wild‐type floral phenotype. The 48 transgenic lines can be categorized into four groups morphologically. Group I (19 lines) were flowerless, with all floral buds aborted before floral stage 9 (see Mandel *et al*. (1992) for the definitions of tobacco floral development stages) (Figure 2a,d,e). Group II (5 lines) had some floral buds that lacked stamens and carpels, while the remaining buds were aborted before floral stage 9. Group III (10 lines) were neuter (stamenless and carpel‐less), with all floral buds lacking stamens and carpels (Figure 2b,f,g). Group IV (14 lines) were carpel‐less, all floral buds developing without carpels (Figure 2c,h,j). For Group IV carpel‐less flowers, pollen grain production was less than 20% of wild‐type flowers. Our pollen germination experiment showed that transgenic pollen from the carpel‐less plants was not viable.

### Morphological differences between *rPTAG2I::DT‐A* tobacco flowers correlated with differences in *DT‐A* expression

**Figure 3:**
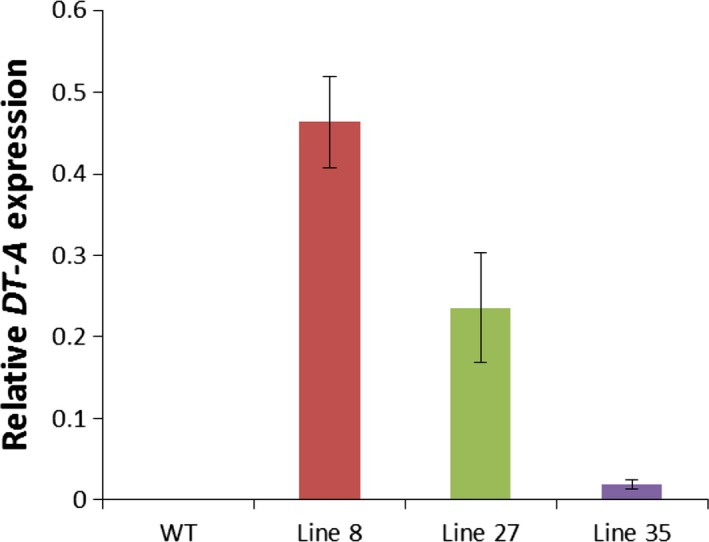
Relative expression levels of the *DT‐A* gene in the 0.7‐mm floral buds (floral stage 6) of three representative *rPTAG2I::DT‐A* tobacco plants, showing that the severity of the floral phenotype correlated with the level of the *DT‐A* gene expression. WT: wild‐type buds; Line 8: flowerless buds from *rPTAG2I::DT‐A*‐Line 8; Line 27: neuter buds from *rPTAG2I::DT‐A*‐Line 27; Line 35: carpel‐less buds from *rPTAG2I::DT‐A*‐Line 35. Expression levels of the tobacco elongation factor 1α gene in each biological replicate were used as an internal reference (Schmidt and Delaney, 2010). Data represent means from three independent biological replicates. Bars show standard errors.

We performed a quantitative real‐time PCR (qRT‐PCR) analysis to confirm the relationship between *DT‐A* expression and flower ablation phenotypes observed in *rPTAG2I::DT‐A* tobacco plants. Approximately 0.7 mm long floral buds, corresponding to floral stage 6 (Mandel *et al*., 1992) were used from representative plant lines: Line 8 (flowerless, floral buds dropped before stage 9), Line 27 (neuter, floral buds having no stamens or carpels), and Line 35 (carpel‐less, with no carpels). Figure 3 shows that *DT‐A* expression was detected in floral buds from all three lines, with a relatively high level in Line 8, a medium level in Line 27, and a low expression level observed in Line 35. A relatively high *DT‐A* expression level in the floral buds of Line 8 plants correlates with the flowerless phenotype, the strongest phenotype observed in the *DT‐A* transgenic lines. A relatively low expression level observed in Line 35 also correlates with relatively normal floral development except ablation of carpels in these plants. A medium expression level detected in the floral buds of Line 27 plants may explain why these plants had an intermediate phenotype between Lines 8 and 35: ablation of both stamens and carpels. We therefore suggest that the expression level of the *DT‐A* gene in floral buds correlate with the severity of the phenotype observed in floral organ ablation.

### Floral ablation phenotypes were stably maintained in vegetatively propagated *rPTAG2I::DT‐A* tobacco progeny under glasshouse and field conditions

To examine the stability of floral ablation phenotypes in vegetatively propagated progeny plants, two representative tobacco lines, Line 8 (flowerless) and Line 27 (neuter, stamenless and carpel‐less), were used for vegetative propagation and further evaluation. As shown in Table 1, all tested plants from vegetatively propagated Line 8 and Line 27 maintained their floral phenotypes respectively under glasshouse conditions. We also planted vegetatively propagated Line 8 and Line 27 plants in the field during the summers of 2014 and 2015. Table 1 shows that in both years, all tested plants from Line 8 maintained their flowerless phenotype and all plants from Line 27 maintained their neuter phenotype. These results demonstrate that flowerless and neuter phenotypes can be maintained in vegetative propagated progeny under both glasshouse and field conditions.

**Table 1:** Characterization of floral morphologies of *rPTAG2I::DT‐A* transgenic tobacco plants in glasshouse and field conditions

Line^a^	No. of plants tested	Normal flower plants	Flowerless plants^b^	Neuter plants^c^	Carpel‐less plants^d^
Glasshouse					
Wild type	20	20	0	0	0
Line 8	23	0	23	0	0
Line 27	19	0	0	19	0
Field					
Year 2014					
Wild type	12	12	0	0	0
Line 8	12	0	12	0	0
Line 27	12	0	0	12	0
Year 2014					
Wild type	12	12	0	0	0
Line 8	12	0	12	0	0
Line 27	12	0	0	12	0

a Line 8 had all floral buds aborted before floral stage 9, which was a representative flowerless plant; Line 27 had all floral buds with no stamens or carpels, which was a representative neuter plant.

b Flowerless plant: all floral buds were aborted before reaching floral stage 9.

c Neuter plant: all floral buds had no stamens or carpels.

d Carpel‐less plant: all flowers had no carpels.

We examined 60 flowers for each wild‐type plant and 100–120 floral buds/ flowers for each transgenic plant.

### Field‐grown flowerless tobacco showed an enhanced biomass production compared to wild‐type plants

**Figure 4:**
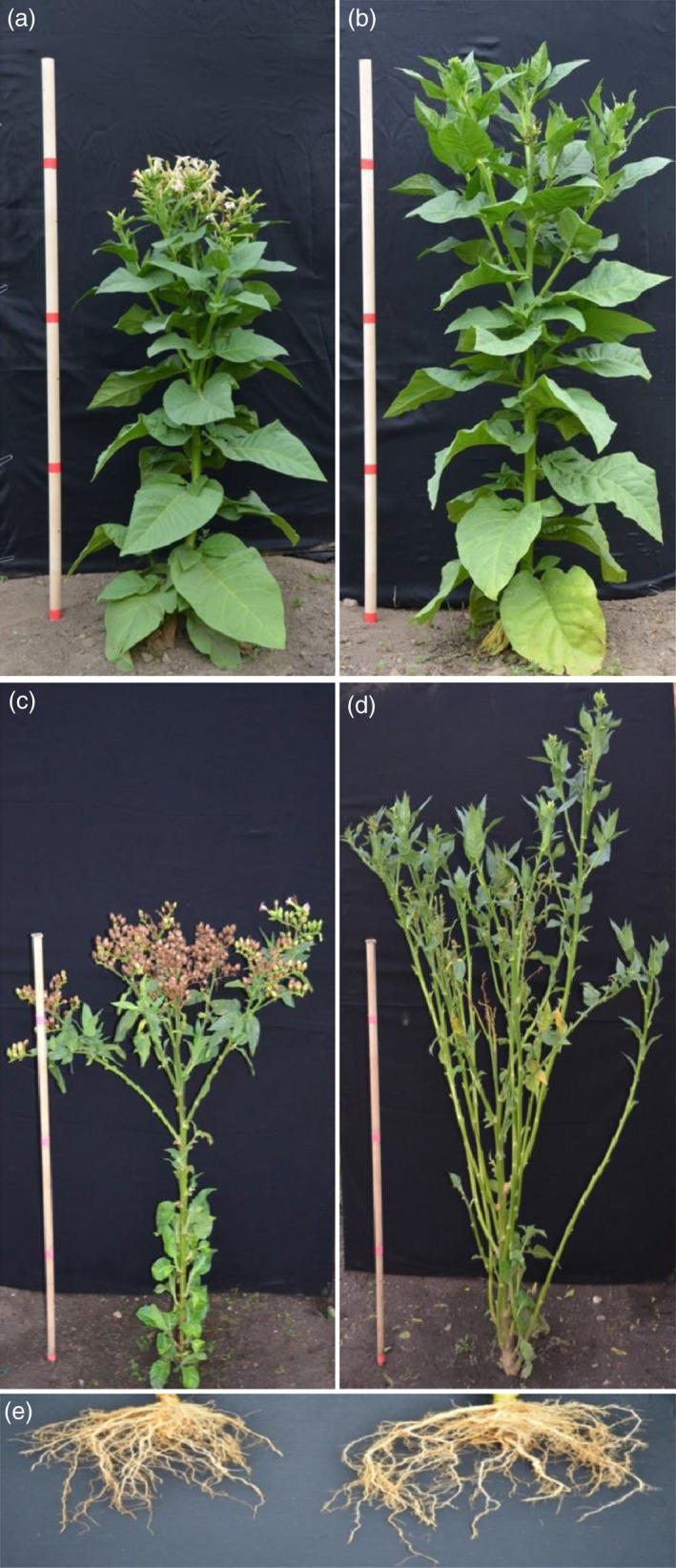
Performance of flowerless transgenic tobacco plants under field conditions. (a) Flowers developed from a 3‐month‐old wild‐type plant. (b) No flowers observed in a 3‐month‐old flowerless plant (*rPTAG2I::DT‐A*‐Line 8) because all floral buds were aborted before floral stage 9. (c) Abundant seeds/seed pods produced from a 4‐month‐old wild‐type plant. (d) No seeds/seed pods produced but more branch shoots developed from a 4‐month‐old flowerless plant (*rPTAG2I::DT‐A*‐Line 8). (e) A better root system (in right) observed in a 4‐month‐old flowerless plant (*rPTAG2I::DT‐A*‐Line 8) when compared to that of a wild‐type plant (in left).

Both Line 8 (flowerless) and Line 27 (neuter, stamenless and carpel‐less) exhibited similar growth as wild‐type plants before flowering (Table 2). For the flowerless plants, the floral meristems failed to develop further when reaching reproductive stages (Figure 4a,b). After growth of floral meristems ceased, lateral shoots of the flowerless plants were released. Flowerless plants developed significant more lateral shoots than wild‐type plants at the end of the growing season (Figure 4c,d). As shown in Table 2, flowerless plants had 72%–91% more dry shoot biomass, and 97%–139% more dry root biomass than wild‐type plants at the end of each growing season, respectively. Similarly, neuter (stamenless and carpel‐less) plants had 91%–115% more dry shoot biomass, and 124%–130% more dry root biomass than wild‐type plants, respectively (Table 2). Dry root/shoot biomass ratios of either flowerless plants or neuter plants were not significantly different compared to those of wild‐type plants. These results demonstrate that under field conditions in 2014 and 2015, the *rPTAG2I::DT‐A* gene did not affect vegetative growth of flowerless tobacco plants before flowering, and enhanced shoot biomass production in the reproductive stages.

**Table 2:** Growth characteristics of field‐grown *rPTAG2I::DT‐A* tobacco plants in 2014 and 2015

Line	Height at flowering (cm)^a^ (mean ± SE)	Height at harvesting (cm)^b^ (mean ± SE)	No. of shoots released (mean ± SE)	Dry shoot biomass (g)^c^ (mean ± SE)	Dry root biomass (g)^d^ (mean ± SE)	Dry root: shoot biomass ratio (mean ± SE)
Year 2014						
Wild type	99.06 ± 2.93	112.18 ± 1.85	6.25 ± 0.75	80.53 ± 8.18	26.53 ± 3.27	0.33 ± 0.011
Line 8 (flowerless)	103.33 ± 4.58	128.69 ± 2.24*	12.0 ± 1.22*	138.73 ± 7.62*	52.17 ± 3.61*	0.38 ± 0.019
Line 27 (neuter)	101.60 ± 1.47	122.77 ± 3.05*	11.25 ± 1.31*	153.60 ± 17.07*	61.13 ± 7.88*	0.40 ± 0.023
Year 2015						
Wild type	86.51 ± 2.87	129.54 ± 6.96	9.50 ± 1.55	142.35 ± 12.59	43.47 ± 3.76	0.31 ± 0.026
Line 8 (flowerless)	90.17 ± 3.20	158.12 ± 1.91*	17.0 ± 1.73*	272.07 ± 7.47*	104.11 ± 3.65*	0.38 ± 0.024
Line 27 (neuter)	84.29 ± 3.42	156.63 ± 4.48*	19.16 ± 1.62*	305.48 ± 9.8*	97.22 ± 11.51*	0.32 ± 0.036

a Height at flowering: plant height when just starting flowering.

b Height at harvesting: plant height when harvesting.

c Dry shoot biomass includes all stem and branch biomass above root collar excluding foliage.

d Dry root biomass includes all root biomass below root collar.

Data were collected from 12 replicate plants and presented as averages. Asterisks represent a significant difference when compared to wild type in the same year using two‐tailed Student's *t* test (*P *≤ 0.05). SE, standard error.

### *rPTAG2I::DT‐A* transgenic poplar plants exhibited similar vegetative growth to the wild type under glasshouse and field conditions

**Figure 5:**
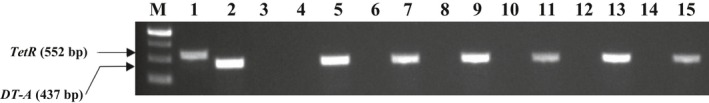
PCR confirmation of stable incorporation of the *rPTAG2I::DT‐A* gene into the genome of representative poplar plant lines used for the field evaluation. PCRs were performed as described in the Experimental Procedures section with primer sequences for the *DT‐A* gene within the T‐DNA region and for the *tetR* gene within the backbone of the Ti‐plasmid, using genomic DNA isolated from representative putative transgenic poplar plants as templates. The Lane M: molecular weight marker. Lanes 1 and 2: the *rPTAG2I::DT‐A* Ti‐plasmid as template with the *tetR* primers (Lane 1) and *DT‐A* primers (Lane 2). Lane 3: Wild‐type poplar plant DNA as template with the *DT‐A* primers. Lanes 4–5, 6–7, 8–9, 10–11, 12–13 and 14–15 are for PCR products using genomic DNA isolated from putative *rPTAG2I::DT‐A* transgenic poplar lines 2, 16, 29, 36, 45 and 57, respectively, with the *tetR* primers for even numbers and the *DT‐A* primers for odd numbers. The presence of the *DT‐A* gene and the absence of the *tetR* gene in the putative transgenic poplar lines demonstrate that these lines should be transgenic. On the other hand, the presence of both the *DT‐A* gene and the *tetR* gene indicates that the genomic DNA from that putative transgenic poplar plant is contaminated with the Ti‐plasmid DNA, and thus, the presence of the *DT‐A* gene does not necessarily support that the plant is transgenic.

We have produced a total of 62 independent poplar lines from two transformations, one in 2014 (27 lines) and the other in 2015 (35 lines) using the *rPTAG2I::DT‐A* gene (Figure 5). These plants were grown under glasshouse conditions and their stem heights and basal stem diameters (2 cm above soil) were measured after 2 months growth. We estimated their biomass index using (BI: height × diameter^2^) according to the method by Wei *et al*. (2007). Our *t*‐test analysis showed that neither the average heights nor biomass indices were negatively affected for the transgenic poplar plants from the 2014 transformations (*P* = 0.7049 for the average heights and *P* = 0.8019 for the biomass indices, respectively). There were also no negative effects observed for the ones produced in the 2015 transformations (*P* = 0.7773 for the average heights and *P* = 0.8565 for the biomass indices, respectively) (Figure 7a,b). On the other hand, we did observe two transgenic polar lines (Line 36 and Line 45) displayed significant reductions in both height growths (25% and 13%, respectively). We have performed a qRT‐PCR assay to determine the expression levels of the *DT‐A* gene in young shoots of these two poplar lines as well as four representative lines with normal vegetative growth. We observed very low but detectable levels of the *DT‐A* gene expression in both Line 36 and Line 45 for which we observed reduction in stem height growth. We also observed no detectable *DT‐A* expression in the four transgenic lines with normal vegetative growth (Table 3).

**Table 3:** Relative expression levels of the *DT‐A* gene in representative *rPTAG2I::DT‐A* transgenic poplar lines

Lines	Relative expression of *DT‐A* gene^a^
Wild type	Not detectable
Line 2	Not detectable
Line 16	Not detectable
Line 29	Not detectable
Line 36	2.1%
Line 45	Less than 0.1%
Line 57	Not detectable

a Relative expression of the *DT‐A* gene in each sample was determined and calculated using the expression level of the *DT‐A* gene in the same tissue sample versus that of the *UBQ* gene. For instance, the *DT‐A* expression level in *rPTAG2I::DT‐A* transgenic poplar Line 36 was 2.1% of the *UBQ* gene.

**Figure 6:**
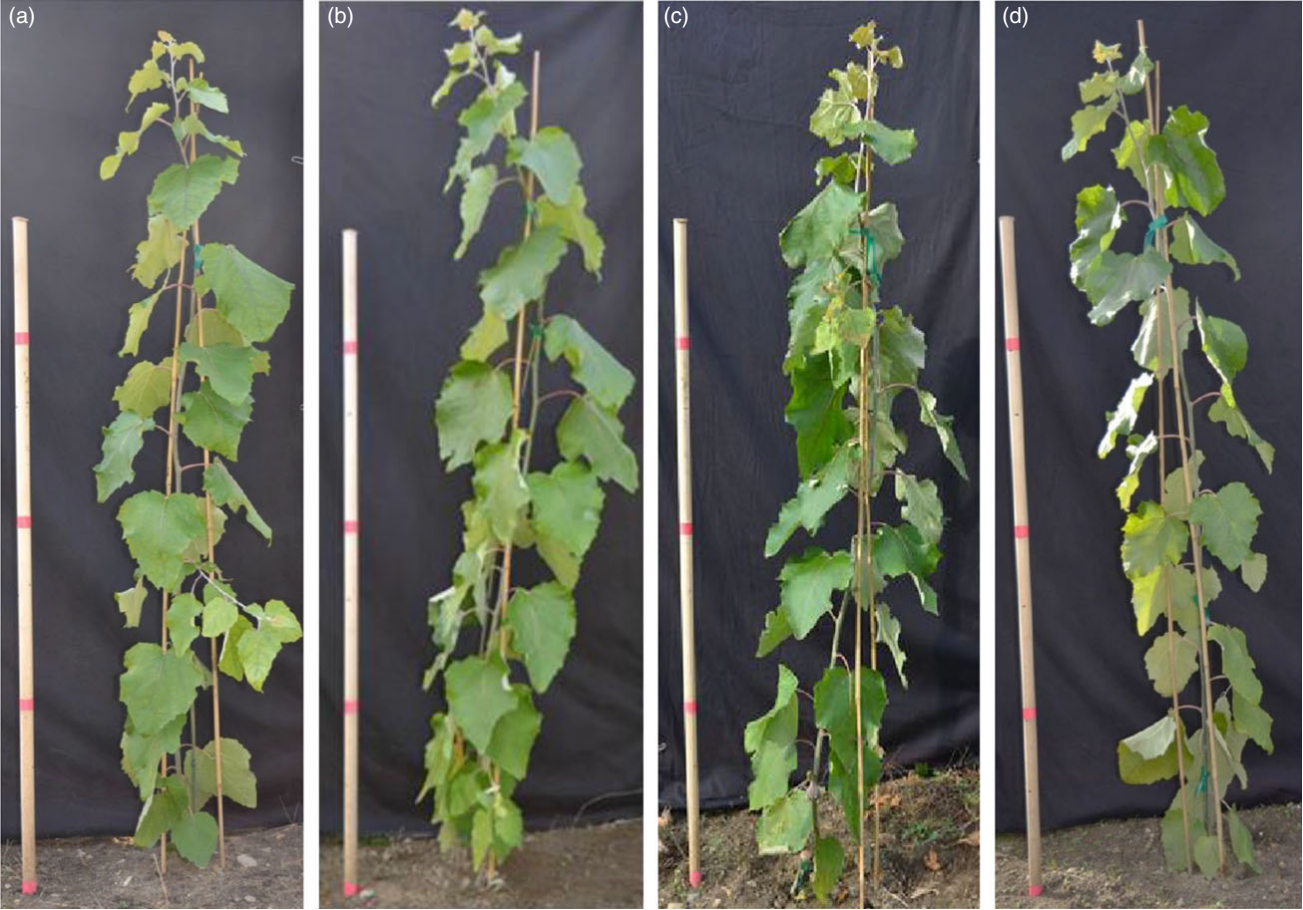
Representatives of *rPTAG2I::DT‐A* transgenic poplar plants grown under field conditions. (a–d) No morphological differences were observed between transgenic (c, d) and wild‐type poplar plants (a, b) after 1‐month growth in the field.

**Figure 7:**
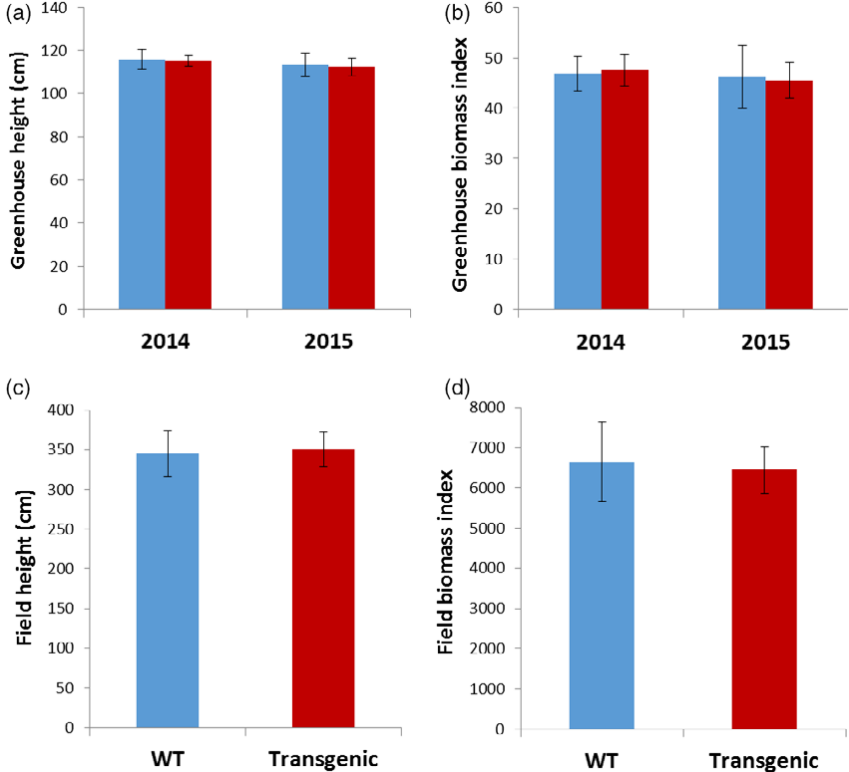
Performance of *rPTAG2I::DT‐A* transgenic poplar plants under glasshouse and field conditions. (a–d) No significant differences in height or biomass index (estimated using height × diameter^2^) between WT and transgenic plants were observed after 2‐month growth in glasshouse for both 2014 and 2015 transformations (a, b), or after two growing seasons in field for 2014 transformation (c, d) according to Student's *t*‐test with the pooled variance at *P *= 0.05. Brackets represent 95 % confidence intervals. Blue bars show data of wild‐type poplar plants and red bars show data of *rPTAG2I::DT‐A* transgenic poplar plants.

We also planted the 27 *rPTAG2I::DT‐A* poplar plants derived from the first transformation in 2014 under field conditions. After two growing seasons in field, we measured the height and diameter of all 27 *rPTAG2I::DT‐A* poplar plants and the wild‐type control plants at the end of November 2015. The average height of transgenic plants was similar to that of the wild‐type plants (*P* = 0.7778) (Figures 6 and 7c). There were no significant differences in the estimated field biomass indices between the transgenic and wild‐type plants (*P* = 0.7078) (Figure 7d). These findings show that no significant differences (*P* > 0.05) were found between wild‐type and transgenic poplar for all variables tested. Depending on the variable, growth differences from 4 to 17% could be tolerated before significance would have been declared under the conditions of our experiments.

## Discussion

In this study, we demonstrated that the reverse orientation of the second intron sequence of poplar *PTAG2* gene, when fused with the minimal *35S* promoter (*rPTAG2I*) confers floral‐specific activity as shown with expression of the β‐glucuronidase gene (*uidA*) in tobacco. We also showed that the expression of the *rPTAG2I::DT‐A* gene in tobacco produced a flowerless phenotype and that the phenotype was maintained under field conditions over two growing seasons in vegetatively propagated progeny plants. We further demonstrated that under field conditions, transgenic tobacco plants expressing the *rPTAG2I::DT‐A* gene produced normal vegetative growth before flower induction, and exhibited increases in vegetative growth during flowering and fruiting stages. Similar to tobacco, we found that in poplar the activity of the *rPTAG2I* promoter was not detectable in all *rPTAG2I::GUS* transgenic lines. Further, we observed that the majority of transgenic poplar plants hosting the *rPTAG2I::DT‐A* gene grew and developed normally relative to wild‐type plants, under both glasshouse and field conditions. The transgenic poplar plants in this study have not reached reproductive maturity and therefore could not yet be evaluated for floral‐specific expression of the *uidA* or *DT‐A* genes. However, we believe that the *rPTAG2I* promoter should be floral dominant in poplar and the *rPTAG2I::DT‐A* gene should produce the flowerless phenotype with little side effects on vegetative growth, because of the following reasons: (i) no significant negative effects on the biomass production were observed in flowerless transgenic tobacco under both field and glasshouse conditions; (ii) similar to *rPTAG2I::GUS* tobacco, no detectable *rPTAG2I::GUS* activity in vegetative organs of poplar plants was observed; (iii) and no significant negative effects were observed in biomass production for the 27 field‐grown *rPTAG2I::DT‐A* poplar lines. However, final confirmation will be obtained after several years when the transgenic poplar plants reach reproductive maturity.

It has been previously reported that the second intron of *Arabidopsis AG (AG)* can direct carpel‐ and stamen‐specific expression of *AG* due to its cis elements (Deyholos and Sieburth, 2000; Sieburth and Meyerowitz, 1997). Bioinformatics analysis of the poplar *PTAG2 *second intron has shown that it contains a list of flower‐related cis‐regulatory elements, such as LFY‐binding sites and a repeat sequence of CCAATCA, which have been demonstrated to maintain *AG* expression in *Arabidopsis* flowers (Busch *et al*., 1999; Hong *et al*., 2003). It has been reported that *PTAG2* gene exhibited floral expression with weak vegetative expression in poplar (Brunner *et al*., 2000). Our study has shown that forward orientation of the *PTAG2* second intron (*fPTAG2I*) exhibited activity mainly in floral organs, as well as in some vegetative organs and tissues, consistent with the observations by Brunner *et al*. (2000). It is, however, not known whether the *PTAG2* second intron sequence plays a role in the expression pattern of the endogenous *PTAG2* gene in poplar.

In contrast to the forward orientation of the *PTAG2* second intron (*fPTAG2I*), reverse orientation (*rPTAG2I*) conferred floral expression with no detectable activity in vegetative organs and tissues. The expression differences between genes driven by either the *fPTAG2I* or *rPTAG2I* promoter reveal that the activity of the poplar *AG* second intron is orientation‐dependent. One possible explanation for this difference is that the cis‐regulatory elements conferring vegetative tissue expression might be closer to the transcription starting site in the *fPTAG2I* sequence, while the same cis elements in the *rPTAG2I* is farther from the transcription starting site.

Highly variable abiotic and biotic conditions in the field could significantly affect transgene expression levels and patterns and therefore the transgenic phenotypes. Remarkable differences of transgene expression or phenotype between field‐ and glasshouse‐grown plants have been reported (Anand *et al*., 2003; Brandle *et al*., 1995; Sharp *et al*., 2002). The flowerless trait has been successfully engineered in a variety of annual plant species (Kobayashi *et al*., 2006; Wang *et al*., 2008; Yang et al., 2010); however, in all cases these transgenic lines were evaluated only under glasshouse conditions and not in the field. Our study demonstrates that the transgene‐mediated flowerless phenotype in tobacco plants can be maintained under field conditions over two growing seasons with no observable adverse effects on vegetative growth. Similarly, we did not observe negative effects on vegetative growth in the majority of the transgenic poplar plants that were engineered with a floral bud toxin gene (the *rPTAG2I::DT‐A*). Further, our study demonstrated that engineering a flowerless phenotype in plants could lead to more biomass production. This is most likely due to the reduction in photosynthate partitioning towards generative development, relative to wild‐type plants, under floral induction conditions. Enhanced biomass production as a result of the flowerless trait could be useful in various energy crops (Kalluri *et al*., 2014; Poovaiah *et al*., 2015; Torney *et al*., 2007).

It has been reported that vegetative growth is very sensitive to toxin gene expression and even low expression levels of a toxin gene in vegetative organs could be detrimental to the growth of plants (Lännenpää *et al*., 2005; Lemmetyinen *et al*., 2004). Also, Wei *et al*. (2007) reported that all of 59 transgenic poplar lines expressing a *LEAFY* promoter::barnase gene had a substantially reduced growth rate after one or two growing seasons under field conditions, even though the growth of some of the same plant lines was similar to that of the wild‐type control plants in the glasshouse. In this study, we found that none of the 27 *rPTAG2I::DT‐A* poplar plants tested under field conditions exhibited observable retarded vegetative growth over two growing seasons, suggesting that *rPTAG2I* promoter is floral dominant with little activities in vegetative organs. However, we did also observe that two of the 35 transgenic poplar lines produced in the 2015 transformation had significant reduction in vegetative growth under the glasshouse conditions when compared to the average stem height of the wild‐type controls. Based on the results of qRT‐PCR, we believe that the reductions in growth of these two transgenic poplar lines are due to the expression of the *DT‐A* gene in stem tissues.

We also tested 25 *rPTAG2I::GUS* lines in the field, and none of them had any detectable GUS activity in vegetative organs. Based on all data from the tobacco and poplar studies, we concluded that *rPTAG2I::DT‐A* could be a useful tool for engineering flowerless poplar plants with normal vegetative growth characteristics. Although the *t*‐test results show that there are no significant differences in vegetative growth between the wild‐type control and *rPTAG2I::DT‐A* transgenic poplar, the number of the independent transgenic poplar lines used for the analysis is relatively small. It is therefore possible that with more transgenic plant lines used, our current conclusion, no significant differences in vegetative growth between the wild‐type and *rPTAG2I::DT‐A* transgenic poplar, may not be valid.

While the flowerless phenotype can be used to reduce concerns over transgene flow, neuter (stamenless and carpel‐less) phenotype and carpel‐less phenotype can have other applications. Both the neuter and the carpel‐less phenotypes would be useful for reducing seed‐mediated invasiveness of some exotic ornamental plants, such as purple loosestrife (Brown *et al*., 2002). Moreover, pollination has been shown to shorten floral duration in a variety of plant species by triggering a number of developmental events, including pigmentation changes, ultimately resulting in petal senescence (Martini *et al*., 2003; Proctor and Harder, 1995; Stead, 1992; Weber and Goodwillie, 2007). Xu and Hanson (2000) reported that incompatible pollination could drastically increase flower longevity in petunia when compared to compatible pollination. Pollination will not occur in the carpel‐less transgenic plants, which can be of value for increasing flower longevity of many ornamental plants.

Although toxin genes as shown here can be effective to produce sterile transgenic plants, commercial use of these genes in transgenic plants may cause concerns (Millwood *et al*., 2015). On the other hand, RNAi or CRISPR techniques that can silence or mutate endogenous genes may offer an alternative tool if abolishing function(s) of an endogenous gene(s) can effectively lead to flowerlessness or sterility. In addition to driving the *DT‐A* gene expression, the *rPTAG2I* promoter also could be used to control expression of genes important for flower or fruit development. For example, cytokinins have been shown to regulate flower size in Petunia (Verdonk *et al*., 2008). However, it has been reported that constitutive up‐ or down‐regulation of cytokinin would negatively impact plant growth and development (Li *et al*., 1992; Werner *et al*., 2008). The *rPTAG2I* promoter could be used to specifically drive the expression of a cytokinin biosynthetic or degradation gene in floral buds or floral organs, which would either increase or reduce flower size with no adverse effects of the growth and development of vegetative organs.

## Experimental procedures

### Isolation and cloning of the *P. trichocarpa AG* 2 (*PTAG2*) second intron fragments

Total genomic DNA was extracted from leaves of *P. trichocarpa* genotype ‘Nisqually‐1′ grown in a glasshouse using a modified CTAB method (Porebski *et al*., 1997). Two hundred nanograms of genomic DNA was used as template for amplifying a 4‐kb second intron from *PTAG2*, one of two *AG* orthologues in *P*. *trichocarpa*, using primer pair PTAG2F3154 (5′‐GTATATACTTAGTTCCTCGGCT‐3′) and PTAG2R7035 (5′‐CTGCGCATTCATGTCATCATTT‐3′). These primers were designed to precisely flank the splice junctions of the *PTAG2* second intron sequence (Brunner *et al*., 2000; GenBank Accession No. AF052571). The amplification condition was as follows: an initial denaturation step at 98 °C for 5 min, followed by 35 cycles of 98 °C for 10 s, 60 °C for 5 s, and 72 °C extension plus a final extension at 72 °C for 10 min. The amplified fragment was cloned into the pGEM‐T easy vector and verified by DNA sequencing.

### Plasmid construction

**Figure 8:**
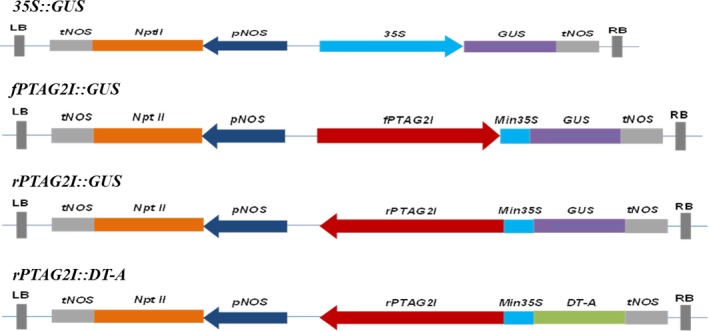
Gene constructs used for tobacco and poplar transformation. LB: left border sequence of T‐DNA. RB: right border sequence of T‐DNA. *tNOS*: nopaline synthase terminator. *NptII*: neomycin phosphotransferase gene. *pNOS*: nopaline synthase gene promoter sequence. *35S*: cauliflower mosaic virus *35S* gene promoter sequence. *GUS*: the coding sequence for the β‐glucuronidase gene. *fPTAG2I*: the forward orientation of the second intron of *P. trichocarpa AGAMOUS (AG) 2* gene. *Min35S*: 60 basepairs of the *35S* gene leader and promoter sequence that has no promoter activity. *rPTAG2I*: the reverse orientation of the second intron sequence of the *P. trichocarpa AGAMOUS* (*AG*) *2* gene. *DT‐A*: the coding sequence for the Diphtheria toxin A (*DT‐A*) gene, which codes for a ribosome inactivating protein.

The amplified second intron fragment of *PTAG2* was first fused with the 60‐bp minimal 35S promoter at the 5′ end in forward and reverse directions to create chimeric promoters of forward orientation (*fPTAG2I*) and reverse orientation (*rPTAG2I*), respectively. The two chimeric promoters were inserted upstream of the *GUS* coding sequence in a pBIN19 vector to create constructs of *fPTAG2I::GUS* and *rPTAG2I::GUS*. Similarly, *rPTAG2I* was inserted upstream of *DT‐A* coding region in a pBIN19 vector to create the construct of *rPTAG2I::DT‐A*, as illustrated in Figure 8. The *GUS* gene under the control of the globally active 35S CaMV promoter in a pBIN19 background (*35S::GUS*) was used as a control vector.

### Tobacco and poplar transformation

Plasmid vectors of all built constructs were introduced into *Agrobacterium tumefaciens* strain EHA105 separately and the resulting bacteria were used to transform *Nicotiana tabacum* cv. Xanthi and *Populus tomentosa* Carr. Tobacco leaf disc transformation was performed as described previously (Zheng *et al*., 2007). Leave discs of Chinese white poplar (*Populus tomentosa* Carr.), approximately 0.5 × 0.5 cm, were incubated with A. *tumefaciens* EHA105 (OD_630_ = 0.5) for 10 min, and then transferred onto sterile filter paper to remove excess liquid and bacteria. After 2 days of cocultivation on WPM medium at 28 °C in dark without hormone and antibiotics, infected discs were transferred to callus‐inducing medium containing 2 mg/L BA, 1 mg/L NAA, 150 mg/L timentin and 30 mg/L kanamycin. After 3 weeks of cultivation at 28 °C in a 16‐h photoperiod, leaf discs with induced calli were subcultured on shoot‐inducing medium containing 1 mg/L BA, 0.1 mg/L NAA, 150 mg/L timentin and 40 mg/L kanamycin. Putative transgenic shoots were then transferred to the rooting medium containing 150 mg/L timentin and 15 mg/L kanamycin. The plantlets were transplanted in soil and grown in a glasshouse.

### Molecular confirmation of transgenic plants

Genomic DNA was extracted from leaves of putative transgenic plants using a modified CTAB method (Porebski *et al*., 1997). To avoid contaminations of Ti‐plasmid DNA from Agrobacterium remaining in transgenic plant tissues, the isolated genomic DNA was fractioned on 0.8% (w/v) agarose gel with the related Ti‐plasmid DNA loaded on the side as a reference. Large‐sized genomic DNAs were recovered from the agarose gels to eliminate all Ti‐plasmid DNA from residual Agrobacterium cells, and the purified plant genomic DNAs were used as templates (Chen *et al*., 2006). The primer pair DT‐AF (5′‐CTTCGTACCACGGGACTAAACTGGTTATGT‐3′) and DT‐AR (5′‐AAGTTCTACGCTTAACGCTTTCGCCTGT‐3′) was used to amplify a 437‐bp fragment from *DT‐A* gene within the T‐DNA region of the Ti‐plasmid, and the primer pair TET‐F (5′‐GACGACTGGCGCTCATTTCT‐3′) and TET‐R (5′‐GCATGAAAAAGCCCGTAGCG‐3′) was used to amplify a 552‐bp fragment containing a partial tetracycline resistance (*tetR*) gene within the backbone sequence of pBIN19 outside the T‐DNA region. PCR solution was 20 μL containing 1 × PCR buffer (Takara Bio Inc., Shiga, Japan), 1.5 mm MgCl2, 0.2 mm dNTPs, 0.2 μL e2TAK DNA polymerase (Takara), 0.25 μm of each primer and 500 ng DNA. The amplification started with an initial denaturation step at 98 °C for 5 min, followed by 35 cycles of 98 °C for 10 s, 60–65 °C for 5 s, and 72 °C extension plus a final extension at 72 °C for 10 min.

### Tissue sectioning and histochemical GUS assays

Poplar shoot apices, as well as tobacco hand‐sectioned leaf, stem, shoot apices and floral buds, were incubated in X‐Gluc solution at 37 °C overnight for histochemical GUS staining. Histochemical assays of GUS activity were performed in a solution consisting of 100 mm potassium phosphate buffer, pH 7.0, 10 mm Na_2_EDTA, 0.5 mm K_3_Fe(CN)_6_, 0.5 mm K_4_Fe(CN)_6_, 0.1% Triton X‐100 and 1 g/L X‐Gluc (5‐bromo‐4‐chloro‐3‐indolyl‐β‐d‐glucuronic acid). Subsequent depigmentation was carried out in ethanol to remove chlorophylls and other pigments gradually prior to visual inspection and photographically recorded.

### Pollen germination assays

Pollen germination assays were performed as described previously (Wang and Jiang, 2011). Basically, pollens were removed from stage 11–12 anthers of both wild‐type and transgenic tobacco plants, and incubated on glass slides with pollen germination medium [0.01% boric acid, 1 mm CaCl_2_, 1 mm Ca(NO_3_)_2_·4H_2_O, 1 mm MgSO_4_·7H_2_O, 10% (wt/vol) sucrose, pH 6.5] at 27.5 °C. After 2 h, the pollen germination rates were recorded.

### Quantitative real‐time PCR analysis of *DT‐A* expression in transgenic tobacco plants

Samples for RNA extraction were collected from vegetatively propagated plants per representative flowerless, neuter, carpel‐less and wild‐type tobacco plant lines. More than 20 0.7‐mm floral buds were collected for each replicate. Total plant RNA was extracted using the RNeasy Plant Mini Kit including RNase‐Free DNase set (Qiagen, Valencia, CA) according to the manufacturer's protocol. The iScript^™^ cDNA Synthesis Kit (Bio‐Rad Laboratories, Richmond, CA) was used to synthesize cDNA, and cDNA products were utilized for quantitative real‐time PCR assays using SsoFast^™^ EvaGreen^®^ Supermix (Bio‐Rad Laboratories) on a CFX96^™^ Real‐Time PCR Detection System (Bio‐Rad Laboratories). The primer pair DTA‐F (5′‐GAGTTTATCAAAAGGTTCGGT‐3′) with DTA‐R (5′‐TTCGCCTGTTCCCAGTTATT‐3′) was used for analysis of DT‐A transcripts, and the pair EF1α‐F (5′‐TGAGATGCACCACGAAGCTC‐3′) with EF1α‐R (5′‐CCAACATTGTCACCAGGAAGTG‐3′) was used to amplify cDNA of the internal reference gene, elongation factor 1α. Data were analysed using CFX Manager^™^ software version 2.0. The *DT‐A* gene expression in each sample was normalized using the expression level of the elongation factor 1α gene in the same sample (Schmidt and Delaney, 2010). Three biological replicates were performed with the wild type and each independent transgenic line.

### Evaluation of stability of floral ablation phenotypes in vegetative propagated progeny plants in the glasshouse

Representative flowerless, neuter and wild‐type plant lines were vegetatively propagated and grown in a glasshouse. After growing for 3 months, the tobacco plants started flowering. To determine the floral phenotype of each plant, 100–120 floral buds/flowers were examined for each transgenic plant.

### Two‐year field evaluations of flowerless transgenic tobacco plants

Representative flowerless, neuter and wild‐type tobacco plants were vegetatively propagated and grown in a glasshouse. One‐month‐old plants with heights of approximately 20 cm were planted in the field located in Storrs, Connecticut, USA in July, 2014 and June, 2015. Field test plots employed a randomized design with 12 replicates. Heights of both transgenic and wild‐type tobacco plants were recorded at initial flowering and final harvest at the end of the growing season (October in 2014 and 2015). At harvest, all tobacco plants were carefully dug out and cut just above the root collars. After removing leaves of all plants, shoot materials (above root collars) and root materials (below root collars) were oven‐dried at 70 °C for 10 days and then weighed. Shoot biomass, root biomass and ratio of root:shoot biomass were determined for each replicate. Data were reported as means of all 12 replicates. Means between field‐grown transgenic and wild‐type tobacco plants were compared using two‐tailed Student's *t*‐test with the pooled variance (Steel *et al*., 1997).

### Vegetative growth evaluation of poplar plants under glasshouse and field conditions

Plants of the 62 *rPTAG2I::DT‐A* transgenic poplar lines and 20 wild‐type plants were acclimatized in pots at 25 °C under a 14‐h photoperiod with a humidity of 80% and then transferred to a glasshouse maintained under ambient light cycles (27 lines and 10 wild‐type plants in the year of 2014, 35 lines and 10 wild‐type plants in 2015). Once acclimated, poplar lines were arranged in a randomized block design. After 2 months of growth in glasshouse, height and basal diameter (2 cm above soil) were measured using a ruler and vernier calliper, respectively. Glasshouse biomass indices were estimated using (height × diameter^2^) (Wei *et al*., 2007). After glasshouse evaluation, the 27 *rPTAG2I::DT‐A* transgenic poplar plant lines derived from the 2014 transformation and 10 wild‐type plants were planted in the field located in Storrs, Connecticut in August, 2014 at a spacing of 5 × 5 feet (1.5 × 1.5 m). Transgenic plants and wild‐type controls were randomly planted. Plants were watered as needed until fully established. Height and basal diameter (both in cm) were taken on all poplar plants at the end of the growing season of (November) 2015. Field biomass indices were estimated using (height × diameter^2^) (Wei *et al*., 2007). Data were reported as the mean of all events. Comparisons of means between transgenic and wild‐type poplar plants were conducted using two‐tailed Student's *t*‐test with the pooled variance (Steel *et al*., 1997).

### Quantitative real‐time PCR analysis of *DT‐A* expression in transgenic poplar plants

The two *rPTAG2I::DT‐A* transgenic poplar lines with retarded vegetative growth, four representative *rPTAG2I::DT‐A* transgenic poplar lines with normal growth, and wild‐type plants were vegetatively propagated and grown in glasshouse. Shoot apices of 1.5 cm from the 2‐month‐old glasshouse‐grown poplar plants were collected for RNA isolation. Total plant RNA was extracted using the RNeasy Plant Mini Kit including RNase‐Free DNase set (Qiagen) according to the manufacturer's protocol. The iScript^™^ cDNA Synthesis Kit (Bio‐Rad Laboratories) was used to synthesize cDNA, and cDNA products were utilized for quantitative real‐time PCR assays using SsoFast^™^ EvaGreen^®^ Supermix (Bio‐Rad Laboratories) on a CFX96^™^ Real‐Time PCR Detection System (Bio‐Rad Laboratories). The primer pair DTA‐F (5′‐GAGTTTATCAAAAGGTTCGGT‐3′) with DTA‐R (5′‐TTCGCCTGTTCCCAGTTATT‐3′) was used for analysis of *DT‐A* transcripts, and the pair UBQ‐F (5′‐GTTGATTTTTGCTGGGAAGC‐3′) with UBQ‐R (5′‐GATCTTGGCCTTCACGTTGT‐3′) was used to amplify cDNA of the internal reference gene, ubiquitin. Data were analysed using CFX Manager^™^ software version 2.0. The *DT‐A* gene expression in each sample was normalized using the expression level of the ubiquitin gene in the same sample. Three biological replicates were performed with the wild type and each independent transgenic line.
